# STAT3 Regulates the Type I IFN-Mediated Antiviral Response by Interfering with the Nuclear Entry of STAT1

**DOI:** 10.3390/ijms20194870

**Published:** 2019-09-30

**Authors:** Huanru Wang, Meng Yuan, Shuaibo Wang, Li Zhang, Rui Zhang, Xue Zou, Xiaohui Wang, Deyan Chen, Zhiwei Wu

**Affiliations:** 1 Center for Public Health Research, Medical School, Nanjing University, Nanjing 210093, China; wanghuanru622@163.com (H.W.); YuanMeng940423@163.com (M.Y.); lizhang_515@163.com (L.Z.); MG1735015@smail.nju.edu.cn (R.Z.); zx@smail.nju.edu.cn (X.Z.); dychen_uni@163.com (D.C.); 2 Jinling College, Nanjing University, Nanjing 210089, China; wangshuaibo0607@163.com; 3 Department of Biology, Hong Kong Baptist University, Kowloon Tong, Hong Kong, China; wxiaohui@smail.nju.edu.cn; 4 State Key Lab of Analytical Chemistry for Life Science, Nanjing University, Nanjing 210023, China; 5 Jiangsu Key Laboratory of Molecular Medicine, Medical School, Nanjing University, Nanjing 210093, China

**Keywords:** STAT, virus, antiviral response, inflammation, nuclear import, immune regulation

## Abstract

Signal transducer and activator of transcription 3 (STAT3) is a multifunctional factor that regulates inflammation and immunity. Knowledge of its regulatory mechanisms is very limited. Here, we showed that enterovirus 71 (EV71) infection induced the phosphorylation of STAT3 and the expression of its downstream inflammatory regulators. Knockdown of STAT3 with siRNAs significantly restricted viral RNA and protein levels, and also reduced viral titers. With further investigation, we found that importin α family member Karyopherin-α1 (KPNA1) was employed by both STAT1 and STAT3 for their nuclear import. The phosphorylated and un-phosphorylated STAT3 competed with STAT1 for binding to the decreased KPNA1 post infection and repressed downstream ISG expression. STAT3 knockdown alleviated the repressed type I IFN-mediated antiviral response upon infection and led to decreased viral replication. Taken together, our data suggested the role of STAT3 in maintaining the balance of inflammation and antiviral responses in the central nervous system (CNS) upon infection.

## 1. Introduction

STAT3 exhibits dual regulatory activities with respect to various biological or clinical settings. It mediates cell death under physiological conditions [1,2], while in tumor cells, STAT3 activation facilitates cell growth and survival [3,4]. In inflammation, the IL-6-activated STAT3 leads to pro-inflammatory responses while the IL-10-activated STAT3 results in anti-inflammatory responses [5,6,7,8]. STAT3 also exhibits dual regulatory roles during viral infection [9,10]. Some of the viral infections were facilitated by STAT3 since specific inhibitors or siRNAs of STAT3 repressed viral replication [11,12,13]. However, with respect to Vaccinia virus and Influenza A virus, STAT3 knockdown increased viral replication with decreased expression levels of IFN-stimulated genes (ISGs) [14]. Viruses also evolve strategies to manipulate STAT3 function to create favorable replication environments [15], such as decreasing the protein and/or the mRNA levels of STAT3 [16,17], inhibiting the phosphorylation of STAT3 [18], or blocking nuclear translocation of activated STAT3 [19,20,21,22,23]. However, the mechanism of actions by STAT3 during viral infection remains poorly understood.

Hand, foot and mouth disease (HFMD) is a pediatric disease that is caused by enterovirus infection. According to previous research, more than 90% of fatal HFMD cases in China from 2008 to 2012 were associated with EV71 infection [24]. As a neurotropic virus, EV71 infection could induce neurological complications including aseptic meningitis and encephalitis in severe cases. The causal mechanisms of inflammation in the central nervous system (CNS) remain incompletely understood. One possibility is that the neuroinflammation mainly results from the side-effect of the antiviral response executed by CNS resident or peripheral immune cells [25,26,27]. Glia cells are the main immune cells in CNS and play critical roles in virus-related neuropathology [28]. We and others demonstrated that besides neurons, EV71 can also infect glia cells, especially astrocytes in patient autopsies and animal models [29,30]. Study about the modulation and coordination between antiviral response and inflammation in glia cells would help to expand our current understanding of CNS pathogenesis in EV71 patients. 

Once the invading viruses are detected by the host’s pattern recognition receptor (PRR), triggering a network of signaling, Type I IFNs are produced to serve as the first line of host defense against the viruses. Type I IFNs induce phosphorylation of STAT1 and STAT2 through the JAK-STAT pathway [31]. The phosphorylated STAT1 forms heterodimers with phosphorylated STAT2 and translocate to the nucleus, where they bind to IFN-stimulated response element (ISRE) and induce the expression of IFN-stimulated genes (ISGs) [32]. Most of the ISGs inhibit infection by targeting different stages of the viral life cycles, building an extensive antiviral state in host cells [33,34,35].

EV71 was shown to subvert the classical type I IFN-mediated host defense upon infection, either by blocking the type I IFN production or by repressing the ISG expression through various mechanisms [36,37,38]. KPNA1 is the key importin required for the nuclear translocation of phosphorylated STAT1 [39]. In this study, we found that viral infection induced the decay of KPNA1, which was consistent with a previous report [38]. Intriguingly, we found that besides the degradation of KPNA1, another indispensable factor that restricts host antiviral response was the competitive binding of STAT3 to KPNA1 during infection. When we knocked down STAT3 with specific siRNAs, the restricted nuclear translocation of activated STAT1 was largely relieved post infection, leading to a recovered antiviral response by the downstream ISGs.

Our research detailed a multifunctional role of STAT3 in EV71 infected glia cells. On the one hand, EV71 infection induces STAT3 activation in both mice astrocytes and human T98G cells. The activated STAT3 induced expressions of its target genes, most of which are inflammatory regulators. On the other hand, both un-phosphorylated and phosphorylated STAT3 competed with STAT1 for KPNA1 binding upon infection, leading to repressed ISG expressions. The regulation role of STAT3 in our study implies a potential mechanism of the host to protect the CNS from injuries caused by either overactive antiviral response or inflammation during viral infection. 

## 2. Results

### 2.1. EV71 Infection Induces Activation of STAT3

**Figure 1:**
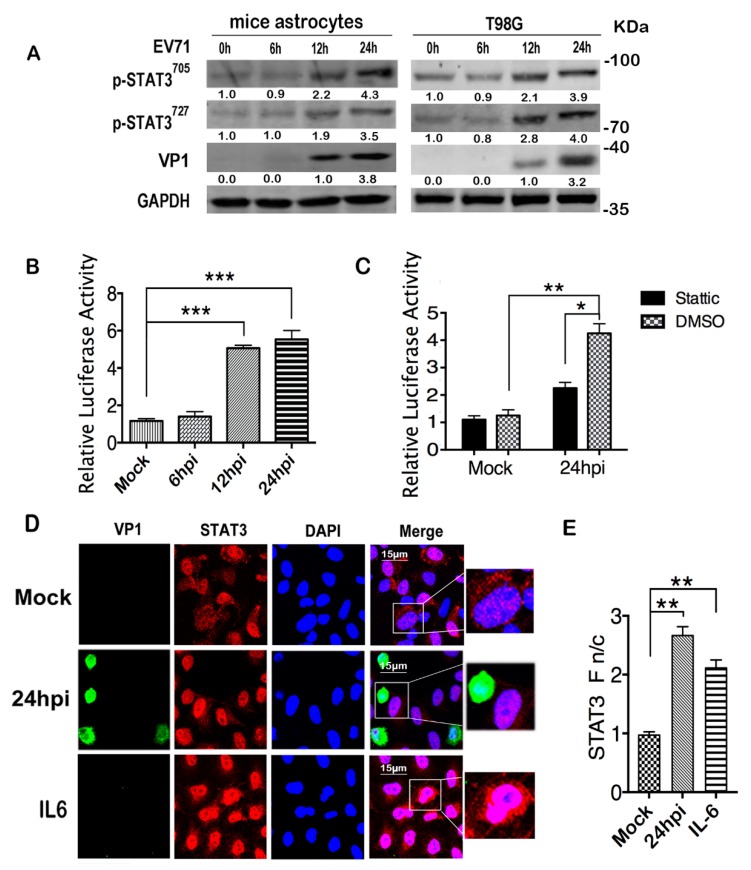
EV71 infection activated STAT3. (**A**) Phosphorylation levels of STAT3 at both Tyr705 and Ser727 sites were increased upon infection. Mice astrocytes and T98G cells were infected with EV71 at a multiplicity of infectivity (moi) of 10. For the mock infection group, culture medium the same as in the viral stock preparation was added with dosage equal to the viral stock in the infected group. Total cell lysates were prepared at indicted time points and subjected to western blot with specific antibodies. Expression levels of indicated proteins were quantified by densitometry scan. The density of each band was first normalized to GAPDH and then to the control (EV71 at time 0 h). A representative result based on three independent experiments is shown. For pSTAT3^705^ and pSTAT3^727^, the value at 0 hpi is set as 1.00 (100%), and for VP1 the value at 12 h post infection (hpi) was set as 1.00 (100%). (**B**) The transcriptional activator activity of STAT3 was increased post infection. T98G cells were co-transfected with pGMSTAT3-Lu along with pGMR-TK (Renilla) as transfection efficiency control; 48h post transfection, cells were infected with EV71 and cell lysates were prepared at indicated time points. The samples were subjected to dual luciferase activity analysis and the values were normalized to pGMR-TK. (**C**) The specificity of the STAT3 transcriptional activator activity reporter system was verified by STAT3 inhibitor Stattic. After co-transfection of pGMSTAT3-Lu and pGMR-TK, T98G cells were treated with Stattic (10 μM) or DMSO for 12h and were infected with EV71. Cell lysates were prepared 24 hpi and measured for dual luciferase activities. (**D**,**E**) EV71 infection induced nuclear translocation of STAT3. T98G cells were infected or mock infected with EV71 and were fixed 24 hpi. Total STAT3 and VP1 were analyzed with specified antibodies by confocal microscopy (**D**). STAT3 Nuclear fluorescence (Fn), cytoplasmic fluorescence (Fc), and background fluorescence (Fb) were analyzed by ImageJ software and calculated using the equation: Fn/c = (Fn − Fb)/(Fc − Fb). Results are from a single assay representative of two or more independent analyses (**E**). Data are shown as the mean ± SEM, *n* > 40. NS, no significance, * *p* < 0.05, ** *p* < 0.01, *** *p* < 0.001.

Glia cells play critical roles in CNS innate immune responses [40,41,42]. While modulation of EV71 infection by host factors was mainly studied in neurons and neuroblastoma cells [43,44], little is known about the regulatory role of glia cells in EV71-related neuropathogenesis. Growing evidence suggests that glia cells are also permissive to EV71 infection [29,30,45]. We infected mice primary astrocytes with EV71 and observed that viral infection induced phosphorylation of STAT3 at both Tyr705 (pSTAT3^705^) and Ser727 (pSTAT3^727^) sites, as detected by antibodies specific for each phosphorylation site (Figure 1A left panel). Human glioma T98G cells were reported to be permissive for EV71 infection [30]. We infected T98G cells with EV71 and, consistent with the mice astrocytes, phosphorylation of STAT3 at both sites was also observed post infection. The transcriptional activator activity of STAT3 was tested with luciferase reporter plasmid pGMSTAT3-Lu in T98G cells; pGMSTAT3-Lu contains STAT3-binding sites inserted upstream of a luciferase gene and controlled by the TK promoter. pGMR-TK vector, with a Renilla luciferase gene controlled by the TK promoter, was used as a control for transfection efficiency. Consistently, we found that the relative luciferase activity was significantly increased post EV71 infection (Figure 1B). As a specific STAT3 inhibitor, Stattic, reported to inhibit the nuclear translocation and activation of STAT3 [46], significantly reduced luciferase activity in the EV71 infected cells, indicating the specificity of the STAT3 luciferase reporter system (Figure 1C). Immunofluorescence (IF) assay showed that in contrast to the mock infected T98G cells in which STAT3 was detected in both the cytoplasm and nucleus, STAT3 was mainly detected in the nucleus of the infection group. Notably, similar to the findings of another group [47], pSTAT3 expression was also detected in the nucleus of VP1 negative cells, suggesting that VP1 expression was not the prerequisite for STAT3 activation. In the IL6-treated cells, which served as a positive control, the concentration of nuclear STAT3 also increased, but unlike the almost undetectable cytoplasmic STAT3 in the EV71-infected cells, the cytoplasmic STAT3 remained detectable (Figure 1D). The Fn/c values of Figure 1D, calculated as described, showed a significant increase in the infected group (Figure 1E). Taken together, our data demonstrated that EV71 infection could induce the phosphorylation of STAT3 in both mice astrocytes and human T98G cells.

### 2.2. STAT3 Targets are Upregulated Early and Downregulated Later during Infection

**Figure 2:**
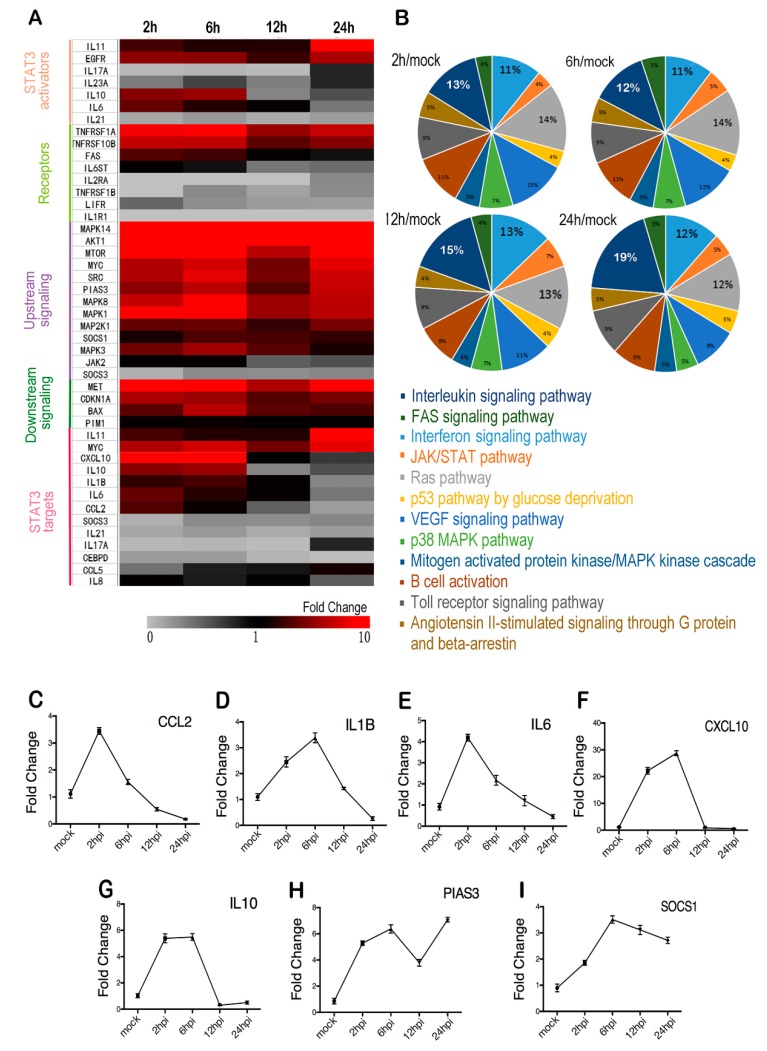
STAT3 signaling-related gene expression during EV71 infection. Total RNA of EV71 infected T98G cells was extracted at indicated time points. After reverse-transcription, the cDNA was subjected to RT2 Profiler PCR Array analysis. (**A**) Relative transcription levels of STAT3-related genes during infection were presented by fold changes in a heat map. In the RT2 Profiler PCR Array, IL-10 and IL-6 were considered to be both activator and targets of STAT3 as shown in the heatmap. (**B**) Differentially expressed genes from each group (log_2_ fold change >2 or <−2, adjusted *p* < 0.05) were subjected to GO Enrichment Analysis and the percentages are shown in pie charts. (**C**–**G**) Fold changes of verified STAT3 targets at indicated time points post infection. (**H**–**I**) Fold changes of STAT3 regulators at indicated time points post infection.

Once phosphorylated, STAT3 dimerizes and translocates to the nucleus and promotes the transcription of its target genes. We performed RT2 Profiler PCR Array analysis with T98G total RNA to investigate the expression profiles of STAT3-related genes during EV71 infection. The genes that are both upregulated by STAT3 activator IL6 and restricted by STAT3 inhibitor Stattic were considered as the target genes of STAT3 in T98G cells (Figure S1). The heat map showed that of all the genes which were significantly increased during EV71 infection; most belong to STAT3 upstream or downstream signaling. While the expressions of STAT3 target genes exhibited a more complicated pattern, despite the immediate decreases of SOCS3, IL21, IL17A and CEBPD from the beginning of the infection (Figure 2A), CXCL10, IL10, IL1B, IL6 and CCL2 exhibited increased expressions during the early infection, and then decreased at 24 hpi (Figure 2C–G). PIAS3 and SOCS1, two regulators that had been reported to restrict the expression of STAT3 target genes [48,49,50], showed a significant increase of the expression upon infection (Figure 2H–I). Genes that had significant changes in expression upon infection were selected and subjected to GO Enrichment Analysis. As shown in the pie charts (Figure 2B), 28 percent of the genes were involved in interleukin signaling pathway and interferon signaling pathway at 12 hpi, and 31 percent at 24 hpi. Due to the high requirement of the regulation in CNS immune response, we speculate that the tightly regulated STAT3 targets contributed to a fine-tuned antivirus status in glia cells to avoid overreaction and tissue damage post viral infection. 

### 2.3. Cellular STAT3 Positively Regulates EV71 Infection

**Figure 3:**
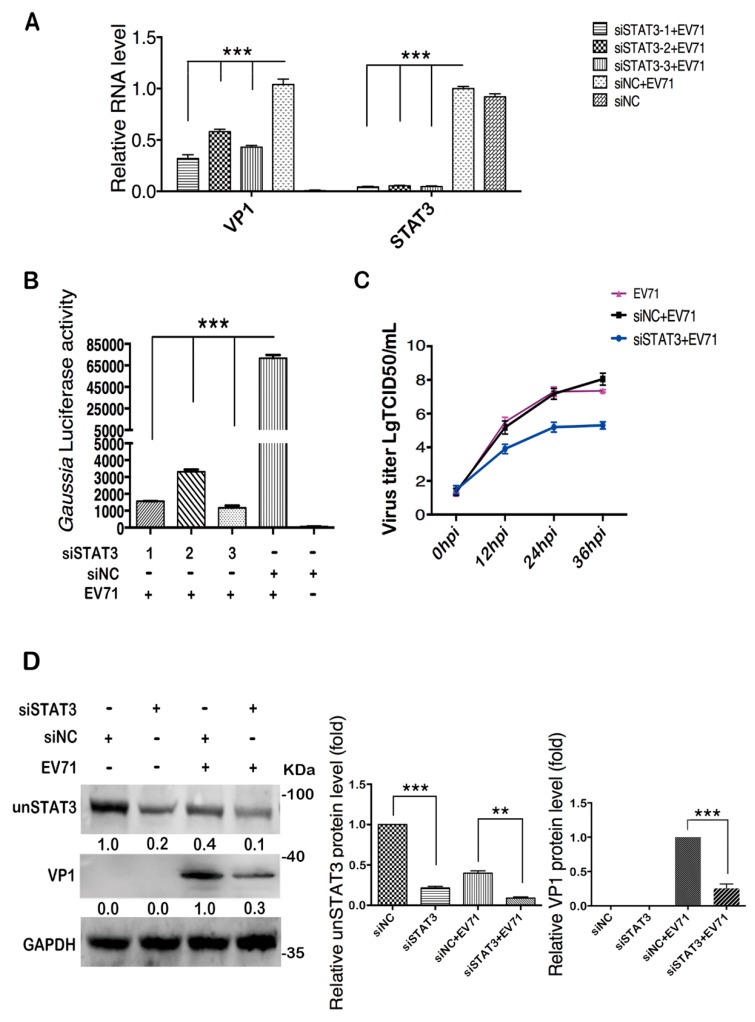
Knockdown of cellular STAT3 restricted EV71 replication. (**A**) STAT3 knockdown restricted viral RNA level. T98G cells were transfected with siRNAs specific to STAT3 (siSTAT3) and were infected with EV71 48 h post transfection. Total RNA was extracted 24 hpi to analyze the RNA level of EV71 and the knockdown efficiency of STAT3. Scrambled siRNA (siNC) was used as a negative control. (**B**) STAT3 knockdown restricted luciferase activity of the EV71 reporter virus. STAT3 knocked down (KD) cells were infected with the EV71 reporter virus (*Gluc*-EV71) with an moi of 15, and cell culture supernatants were harvested 24 hpi, and *Gaussia* luciferase activity was measured. (**C**) Viral titer of EV71 was decreased in STAT3 KD cells. EV71 Brcr strain was used to infect STAT3 KD cells and culture supernatant was collected at indicated time points for TCID_50_ assay. (**D**) EV71 protein level in STAT3 KD T98G cells was analyzed by western blot. STAT3 knockdown efficiency was also determined. Relative expression levels of indicated proteins were calculated as described. For unSTAT3, the value in the siNC group was set as 1.00 (100%), and for VP1, the value in the siNC+EV71 group was set as 1.00. Data are shown as the mean ± SEM of three independent experiments, ** *p* < 0.01, *** *p* < 0.001.

We downregulated STAT3 by specific siRNAs to investigate the role of cellular STAT3 in EV71 infection. Transfection of three siRNAs that target different regions of STAT3 mRNA inhibited STAT3 expression by more than 90% and significantly reduced viral RNA, as compared to the control cells transfected with scrambled siRNA (siNC) (Figure 3A). Reporter virus Gluc-EV71 with a *Gaussia* luciferase gene between the 5′UTR and VP4 gene was employed to quantify the effect of STAT3 knockdown on viral replication. Consistently, the *Gaussia* luciferase activity was significantly reduced after STAT3 knockdown (Figure 3B). Since the three different STAT3 siRNAs all worked well in T98G cells, we used an equal molar mixture of three siRNAs to knockdown STAT3 and determined the effect on viral titers. Cell viability was not affected by STAT3 knock down as assessed by Cell Count Kit-8 (Figure S2). TCID_50_ assay showed that after STAT3 knockdown, EV71 titer was reduced at various time points post infection (Figure 3C). The expression of the EV71 VP1 protein also exhibited pronounced reduction in the STAT3 knockdown T98G cells (Figure 3D). In addition, we observed that in the cells transfected with siNC, the un-phosphorylated STAT3 (unSTAT3) level was significantly decreased upon EV71 infection as compared to the mock infected group (Figure 3D). Taken together, these data suggest that cellular STAT3 plays a positive role in EV71 replication.

### 2.4. STAT3 Knockdown Elevates ISGs Production in EV71 Infected Cells

**Figure 4:**
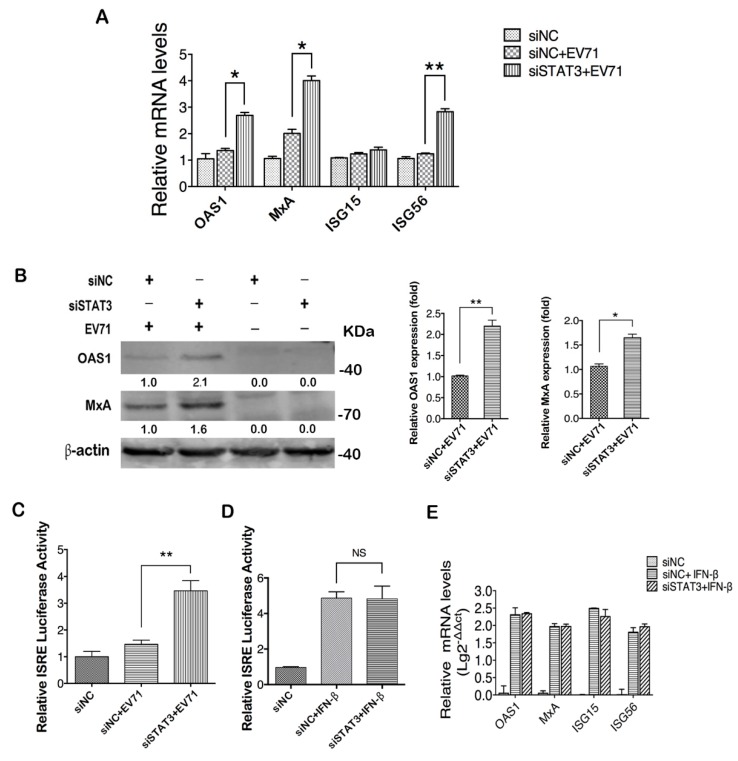
STAT3 knockdown facilitated ISG expression in EV71-infected T98G cells. (**A**) mRNA levels of ISGs from EV71-infected STAT3 KD T98G cells were analyzed by real-time PCR. (**B**) Protein levels of MxA and OAS1 at 24 h post EV71 infection were analyzed by western blot. Relative expression levels of indicated proteins were calculated as described above. The value in the group with siNC transfection and EV71 infection was set as 1.00 (100%). (**C**) STAT3 knockdown increased ISRE activity in EV71 infected cells. Cells were co-transfected with STAT3 siRNA, pISRE-Luc and pGMR-TK; 36h post transfection, the cells were infected with EV71 for 24 h and cell lysates were prepared for dual luciferase activity determination. (**D**) ISRE activity in IFN-βtreated cells was not affected by STAT3 knockdown. Post co-transfection of STAT3 siRNA, pISRE-Luc and pGMR-TK. Cells were treated with IFN-β for 12 h, and dual luciferase activity of the cell lysates was determined. (**E**) Expression levels of ISG mRNAs were not affected by STAT3 knockdown in IFN-βtreated cells. T98G cells with or without STAT3 knockdown were treated with IFN-β and total RNAs were extracted; the mRNA levels of OAS1, MxA, ISG15 and ISG56 were analyzed by real-time PCR, * *p* < 0.05, ** *p* < 0.01.

We further explored the underling mechanism of STAT3 regulation of EV71 infection. As a multifunctional molecule, STAT3 takes part in the regulations of various biological processes including the type I IFN response which is critical for the host defense during viral infection [11,14]. When T98G cells were transfected with STAT3 specific siRNA and infected with EV71, the expression of ISGs, including OAS1, MxA and ISG56 except ISG15, showed a significant increase in their RNA levels (Figure 4A). WB analysis with antibodies specific for OAS1 and MxA showed upregulated expressions of respective proteins after STAT3 knockdown (Figure 4B). Since the expressions of OAS1 and MxA are directly controlled by the interferon-stimulated response element (ISRE), we employed an ISRE luciferase reporter system to analyze the influence of STAT3 knockdown on ISRE activity and showed that the EV71-induced ISRE activity was significantly increased when cellular STAT3 was knocked down, suggesting that STAT3 negatively regulates ISRE activity (Figure 4C). In contrast, IFNβ-induced ISRE activity was not affected by STAT3 knockdown in cells (Figure 4D), suggesting that the restriction of ISRE activity by STAT3 is only associated with EV71 infection. This was further confirmed by real-time PCR analysis that the IFNβ-induced upregulation of ISGs remained unchanged by STAT3 knockdown (Figure 4E). 

To investigate whether the increased ISRE activity and ISG production in STAT3 knockdown cells was due to the increased IFNs in response to EV71 infection, we evaluated expression levels of type I IFNs after STAT3 knockdown. Consistent with a previous study, our result showed steady reduction of the type I IFN expression post EV71 infection [37], and STAT3 knockdown did not change the expression patterns of IFN-α and IFN-β (Figure S3A,B). 

### 2.5. STAT3 Knockdown in Infected Cells Facilitates Nuclear Translocation of STAT1

**Figure 5:**
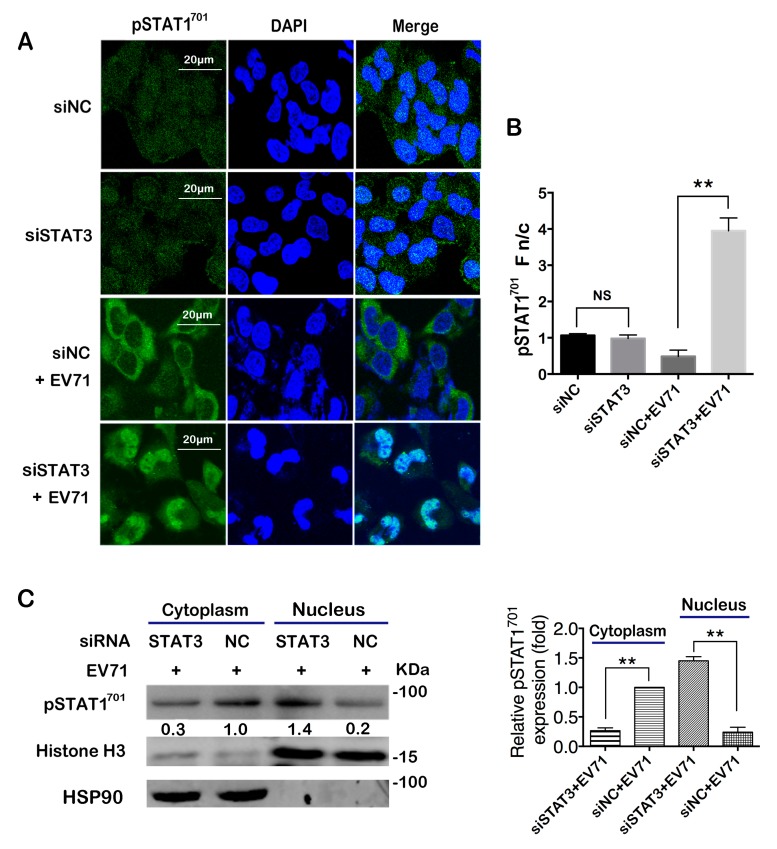
EV71-induced nuclear translocation of STAT1 was reduced in STAT3 knockdown cells. After STAT3 siRNA transfection and EV71 infection, (**A**) immunofluorescence (IF) assay was performed to analyze the effect on STAT1 nuclear translocation in T98G cells. (**B**) Fn/c ratio in (**A**) was determined by ImageJ software as described. (**C**) Nuclear and cytoplasmic proteins were extracted and the protein level of pSTAT1^701^ was determined by western blot. The density of the nuclear protein was first normalized to Histone H3 and then to the control; the cytoplasmic protein was first normalized to HSP90 and then to the control. The value in the siNC+EV71 group was set as 1.00 (100%), ** *p* < 0.01.

STAT1 is a major mediator of the type I interferon response to viral infection [51]. During the process of ISG expression, several steps could affect the ISRE activity, including the phosphorylation of STAT1 and STAT2, the dimer formation, and their nuclear translocation [33]. Since STAT3 knockdown seemed to have no impact on their expressions, we proceeded to analyze the phosphorylation status of STAT1 and STAT2. Our result showed that EV71-induced phosphorylation of STAT1 and STAT2 was not affected by STAT3 knockdown (Figure S4A). The interaction between phosphorylated STAT1 and STAT2 was also not affected as shown by immunoprecipitation (IP) assay (Figure S4B). Remarkably, when nuclear translocation of STAT1 was investigated, we found that although viral infection increased the expression level of pSTAT1^701^, most of the pSTAT1^701^ localized in the cytoplasm instead of the nucleus, but when STAT3 was knocked down by siRNA, a significant amount of pSTAT1^701^ was translocated into the nucleus upon infection (Figure 5A,B). To confirm, we isolated cytoplasmic and nuclear proteins and analyzed them in a WB assay. Consistently, pSTAT1^701^ in the nucleus was significantly increased when cellular STAT3 was knocked down (Figure 5C). These data suggest that the nuclear translocation of pSTAT1^701^ was restricted post infection, and knockdown of cellular STAT3 could alleviate the restriction.

### 2.6. STAT3 Competes with Activated STAT1 for Binding with KPNA1 during Infection

**Figure 6:**
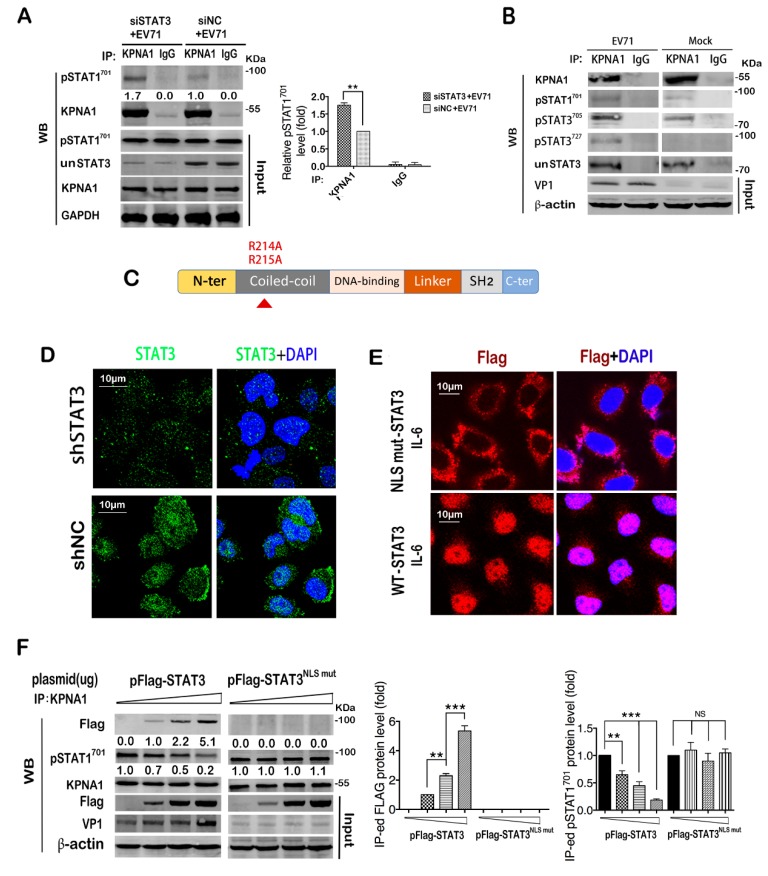
pSTAT1^701^ binding of KPNA1 post EV71 infection was reduced in STAT3 knockdown cells. (**A**) STAT3 knockdown increased the interaction of pSTAT1^701^ and KPNA1. Cell lysates of EV71 infected STAT3 KD cells were immunoprecipitated with an KPNA1-specific antibody and with a normal rabbit IgG as an isotype control. KPNA1-bound pSTAT1^701^ was determined by western blot with a pSTAT1^701^-specific monoclonal antibody. An equal quantity of KPNA1 from siNC+EV71 and siSTAT3+EV71 was loaded and the density of the co-precipitated pSTAT1^701^ protein was analyzed. The value in the siNC+EV71 group was set as 1.00 (100%). (**B**) Binding of pSTAT1^701^, pSTAT3^705^, pSTAT3^727^ or unSTAT3 to KPNA1 12 hpi was determined by IP assay and western blot. (**C**) Schematic diagram of the flag-tagged STAT3 NLS point mutant (STAT3 ^NLS mut^). (**D**) STAT3 knockdown efficiency in T98G cells treated with lentivirus-shSTAT3 (lenti-shSTAT3) was determined by IF assay. (**E**) Nuclear translocation of STAT3 was blocked in STAT3 ^NLS mut^ transfected cells. Lenti-shSTAT3-infected T98G cells were transfected with STAT3 ^NLS mut^ or a flag-tagged wild type (WT) STAT3 plasmid. After treatment with 50 ng/mL IL-6 for 1h, cells were fixed and STAT3 location was detected by the anti-flag antibody in IF assay. (**F**) Increased STAT3 expression level led to decreased binding of KPNA1 and pSTAT1^701^. Lenti-shSTAT3 infected T98G cells were transfected with 0 ug, 5 ug, 15 ug or 25 ug WT STAT3 plasmid and were infected with EV71 36 h post transfection. Cell lysates were prepared at 24 hpi and subjected to IP assay with the KPNA1-specific antibody. Binding of STAT3 to KPNA1 was determined by the anti-flag antibody via western blot. Cells transfected with STAT3 ^NLS mut^ were used as the negative control. Relative protein levels were calculated as described, ** *p* < 0.01, *** *p* < 0.001.

We proceeded to investigate the underling mechanism by which STAT3 regulated the nuclear translocation of pSTAT1^701^. Nuclear import of tyrosine-phosphorylated STAT1 is mainly mediated by KPNA1 (karyopherin subunit α 1), a member of the importin α family [52,53,54]. Consistent with a previous report in RD cells [38], we found that KPNA1 decreased gradually in T98G cells as the infection proceeded (Figure S5); this might be a causative factor for the restricted nuclear translocation of pSTAT1^701^ in the infected cells as shown in Figure 5A,B. To investigate whether STAT3 affects the binding of pSTAT1^701^ to KPNA1, we performed co-IP analysis of pSTAT1^701^ and KPNA1 in STAT3 knockdown cell lysates and showed that knockdown of STAT3 increased the binding of pSTAT1^701^ with KPNA1 in infected cells, as indicated by the increased pSTAT1^701^ co-precipitated with anti-KPNA1 antibody (Figure 6A). STAT3 nuclear import was also reported to be mediated by KPNA1 [55,56,57]. We, therefore, postulate that STAT3 may compete with pSTAT1^701^ for KPNA1 binding for nuclear transportation, thus regulating the downstream effector signaling. To verify this hypothesis, we firstly determined whether STAT3 binds to KPNA1 in T98G cells. The complex immunoprecipited by the KPNA1 antibody was detected with antibodies specific to pSTAT3^705^, pSTAT3^727^ and un-phosphorylated STAT3. The result showed that both the phosphorylated and un-phosphorylated STAT3 could bind KPNA1 in infected cells, suggesting that KPNA1 binding is independent of STAT3 phosphorylation (Figure 6B). Expression levels of these proteins in the immunoprecipitation inputs were also determined by western blot (Figure S6). These data indicate that during EV71 infection, the phosphorylated and non-phosphorylated STAT3 may compete with pSTAT1^701^ for binding to KPNA1 and may affect pSTAT1^701^ nuclear import. 

To further investigate the competition for KPNA1 binding, we constructed a STAT3-NLS mutant (STAT3^NLSm^) which is unable to bind KPNA1 [55], and employed a lentivirus expressing shSTAT3 to reduce the endogenous STAT3 level in T98G cells to an undetectable level to eliminate the interference of endogenous STAT3 (Figure 6C,D, Figure S7). Cells were then transfected with STAT3^NLSm^ or a wild type STAT3 construct (wtSTAT3) for 48h. After IL-6 treatment, the cells were fixed and IF assay was performed with anti-flag antibodies to visualize the localization of exogenous STAT3 (Figure 6E). The result showed that STAT3^NLSm^ was impaired in nuclear translocation and retained in the cytoplasm, in contrast to wtSTAT3 which was predominantly localized to the nucleus after IL-6 stimulation.

Using this system, we performed in vitro competition assay by transfecting the cells with increasing doses of the STAT3 plasmid before EV71 infection and measured the STAT1 and STAT3 binding of KPNA1 by IP assay. It was found that binding of pSTAT1^701^ to KPNA1 was reduced as the wtSTAT3 transfection doses increased with a concomitant increase of VP1 (Figure 6F, left panel), which was not observed with the mutant STAT3^NLSm^ (Figure 6F, right panel), suggesting that STAT3 competes against pSTAT1^701^ for binding to KPNA1. As the competition was mitigated in cells transfected with mutant STAT3^NLSm^, the restored STAT1 activation led to marked decreases in VP1 expression as shown. Taken together, our data demonstrated that STAT3 regulated nuclear import of pSTAT1^701^ by binding with the limited KPNA1 post EV71 infection. 

### 2.7. EV71 Infection Induces STAT3 Degradation via Proteasome Dependent Pathway

**Figure 7:**
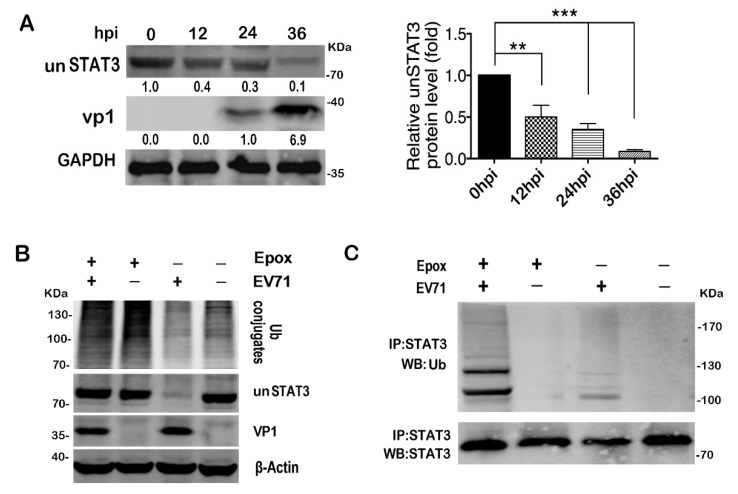
EV71 infection induced proteasome-dependent degradation of un-phosphorylated STAT3. (**A**) unSTAT3 protein levels at indicated time points post infection were determined by western blot. (**B**) Epoxomicin inhibited unSTAT3 degradation post infection. T98G cells were treated with or without 2 μM epoxomicin (Epox) for 2h and were infected with EV71. Cell lysates were prepared 36 hpi, and Ub conjugates as well as unSTAT3 were analyzed with specified antibodies. (**C**) IP assay was performed with STAT3 specific antibody and the precipitants were analyzed with ubiquitin specific antibody, ** *p* < 0.01, *** *p* < 0.001.

A reduced un-phosphorylated STAT3 (unSTAT3) protein level was observed 24h post EV71 infection (Figure 3D). To confirm, we expanded the infection time to 36h by using a low moi of 5 and the protein level of unSTAT3 decreased to 10% of the mock group, but the mRNA level remained constant (Figure 7A, Figure S8A), suggesting a post-transcriptional regulation of unSTAT3 during viral infection. EV71 viral proteins 2A and 3C are well known for their manipulation of host defense by proteolytic cleavage of key cellular factors [37,58,59,60,61]. However, our data did not show any effects of overexpressed GFP-tagged 2A and 3C on the unSTAT3 protein level (Figure S8B,C). However, when the infected cells were pre-treated with epoxomicin (Epox) to inhibit proteasome activity, unSTAT3 was rescued from degradation (Figure 7B), indicating that EV71-induced unSTAT3 degradation was mediated through a proteasome-dependent pathway. Further IP analysis showed that when the proteasome activity was inhibited, the ubiquitinated STAT3 in the infected cells could be clearly detected by ubiquitin-specific antibody (Figure 7C). Due to the positive role of STAT3 in EV71 infection, it was unlikely that the ubiquitin-dependent decay of un-phosphorylated STAT3 was a viral strategy. We speculate that this STAT3 competition may be an evolutionarily conserved mechanism of the host to tightly control the immune response to avoid tissue damage. Taken together, our data demonstrated that EV71-induced unSTAT3 degradation via the ubiquitin-dependent pathway.

## 3. Discussion

EV71 infection causes neuroinflammation including encephalitis and meningitis in severe cases [26,27,62]. Immune and inflammatory responses in the brain need to be subtly regulated to effectively eliminate the pathogen and at the same time keep the neurons from immune injury. Glial cells are the predominant cell type of the immune system in the brain [28,63,64]. Understanding the regulatory mechanism in EV71-infected glial cells will allow us insights into the disease pathogenesis and treatment of the patients. 

In the present study, we found that STAT3 was activated upon EV71 infection in mouse primary astrocytes and human T98G glioma cells. Among its target genes, we observed increased expression of IL6, IL8, CCL5 and CXCL10 at different time points post infection. According to previous studies on cerebrospinal fluid (CSF) chemokines in EV71 patients with encephalitis or meningoencephalitis (ME), CSF IL-6, IL8, CCL5 and CXCL10 concentrations were significantly higher compared to patients with febrile convulsion (FC) [25,26]. Our data suggests that STAT3 signaling in glia cells might participate in the progression of EV71-induced neurogenic pathogenesis.

STAT3 targets were upregulated at the early stage of infection and then mostly depressed at 24 hpi as the infection proceeded, indicating that STAT3 activation in glial cells was under tight control during infection. The protein inhibitor (PIAS3) of activated STAT3 was reported to bind activated STAT3 and inhibit its DNA binding activity, leading to the blockade of STAT3 target gene expression [49]. Suppressor of Cytokine Signaling proteins (SOCS) was reported to interact with Janus-Activated Kinase (JAK) and inhibit STAT signaling upstream [48]. The upregulation of PIAS3 and SOCS1 and the significant degradation of unSTAT3 at the later stage of infection may partially explain the reduced expression of STAT3 target genes in the late stage of EV71 infection. The STAT3 targets that increased in the early infection were mainly inflammatory regulators including pro-inflammatory factors IL1B, IL6 and immunosuppressive factor IL10, which might contribute to the immune regulatory role of glia in CNS during infection.

We found that knockdown of STAT3 reduced EV71 infection in T98G cells, which was correlated with the rescued ISG production. From pathogen-associated molecular patterns (PAMPs) recognition of ISG expression, type I IFN-mediated antiviral response can be regulated at different stages, including IFN induction, STAT1/STAT2 phosphorylation, interferon-stimulated gene factor 3 (ISGF3) formation and its nuclear translocation [33,65]. Growing evidence suggests that STAT3 negatively regulates IFN-mediated antiviral responses since ISRE reporter activity and ISG induction were increased after STAT3 knockdown [11,13]. The detailed mechanism of the negative regulation remains to be fully investigated. 

It is known that the substrates of the nuclear import system prefer importin α isoforms [57,66,67]. Containing a non-classic import signal, STAT1 prefers to bind with KPNA1 (importin α5) at the C-terminus of KPNA1 [52]. KPNA1 is also reported to be the predominant importin for STAT3 translocation with its C-terminus required for STAT3 nucleus import [55]. Our present study demonstrated that translocation of activated STAT1 and STAT3 induced by EV71 infection required KPNA1 in T98G cells, and consistent with a previous study in RD cells [38], we observed degradation of KPNA1 in T98G cells upon infection. The competition between pSTAT1^701^ and STAT3 (both phosphorylated and un-phosphorylated) for KPNA1 binding thus restricts pSTAT1^701^-mediated antiviral response, which is exacerbated by the degradation of KPNA1. When STAT3 was knocked down, the restricted pSTAT1^701^ nuclear translocation was relieved and ISG expression was restored, resulting in the decrease of EV71 replication by type I IFN-mediated antiviral response. Since unSTAT3 was also degraded upon EV71 infection, at least in the current study, the EV71-induced unSTAT3 degradation was not sufficient to block the nuclear translocation of the activated pSTAT1^701^ (Figure 5A) and blocked the downstream anti-viral signaling (Figure 4A). It is plausible that antiviral response is regulated by the relative abundance of STAT3 and KPNA1, which were affected by viral replication. Our data expanded the current understanding on the novel roles of STAT3 in regulating the type I IFN response during viral infection.

Although it was initially believed that nuclear import of STAT proteins requires their tyrosine phosphorylation and dimerization, the nuclear presence of un-phosphorylated STAT3 was also observed by several research groups, and emerging evidence suggests that tyrosine phosphorylation is not a prerequisite for STAT3 nuclear entry [68,69,70]. Consistently, our IP assay showed that both phosphorylated and un-phosphorylated STAT3 can bind KPNA1 (Figure 6B). Further studies are still needed to clarify the KPNA1-binding affinities of the Tyr705 phosphorylated, Ser727 phosphorylated or un-phosphorylated STAT3, as well as their relative contribution in the KPNA1-binding competition. 

We speculate that this STAT3 competition may be an evolutionarily conserved mechanism of the host to tightly control the immune response to avoid tissue damage. When there is robust viral replication, the host cell may drive STAT3 degradation and allow increased STAT1 nuclear translocation to promote the type I IFN response. If the viral replication is restricted, increasing cytosolic STAT3 will compete against STAT1 for binding KPNA1 and reduce the nuclear translocation of activated STAT1, limiting the type I IFN response to avoid overreaction. Similar to the balance between STAT1 and STAT3 in tumorigenesis [71,72], we proposed a balance between STAT1 and STAT3 in the innate immune response during EV71 infection. Taken together, our results showed that STAT3 activation in EV71-infected glia cells leads to upregulation of immune regulatory factors, which were tightly controlled, and revealed the detailed mechanism that STAT3 interfered with type I IFN-mediated antiviral response during viral infection. Further studies on other cells types with different expression levels of STAT1, STAT3 and KPNA1 would provide us more clues to the universality of this STAT3 regulation mechanism in the type I IFN response. Nevertheless, we documented a novel regulatory mechanism by STAT3 in modulating innate immune response to EV71 infection and expanded the scope of the present understanding of the multifunctional roles of STAT3.

## 4. Materials and Methods

### 4.1. Ethics Statement

Animal experimental protocols were approved by the Nanjing University Animal Care Committee (#2014-SR-079, 25 October 2014) and followed the ‘Guide for the Care and Use of Laboratory Animals’ published by the Chinese National Institutes of Health.

### 4.2. Cells and Virus

Mice primary astrocytes were prepared as described previously with modifications [73]. Briefly, 2-3-day-old BALB/c mice were sacrificed, cerebral cortices of 7 mice were isolated and the meninges were removed. The tissue was disaggregated by gently pipetting, and large debris in the dissociated cells were removed by a 70 μm cell strainer. Then, the cells were centrifuged at 800 rpm for 5 min, and transferred to a T-75 culture flask after resuspension with culture medium (DMEM/Ham’s F12 with 4.5 g/L glucose, 15 mM HEPES, 2 mM Glutamine, 10% fetal bovine serum and 1% Penicillin/Streptomycin). Media were replaced every 3 days. Upon reaching 95% confluence, cells were passaged to generate a sufficient cell number.

T98G and Vero E6 cells (ATCC) were maintained in Dulbecco’s modified Eagle’s medium (DMEM) containing 10% fetal bovine serum (FBS), 100 U/mL penicillin and 100 g/mL streptomycin. EV71 BrCr strain, a gift from Professor Bin Wu, Jiangsu Provincial Centers of Disease Control, was propagated in Vero cells, and the 50% tissue culture infectious dose (TCID_50_) was determined on Vero cells as described [74,75]. Gluc-EV71 that carries a Gaussia luciferase reporter gene in the EV71 genome was a kind gift from Bo Zhang, Wuhan Institute of Virology, CAS (Wuhan, China). Gaussia luciferase activity was determined by the Gaussia Luciferase Glow Assay Kit (Pierce, Rockford, IL, USA). shSTAT3-lentivirus was obtained from Santa Cruz Biotechnology (Santa Cruz, CA, USA).

### 4.3. Plasmids, siRNAs, Antibodies and Inhibitors

Plasmid pEGFP-2A was constructed by Ying Chu as previously described [74]. Plasmid pEGFP-3C was a gift from Dr. Xiaobo Lei and Professor Jianwei Wang [76]. Plasmid expressing STAT3 with a flag tag was purchased from Sino Biological (Beijing, china). pSTAT3 ^NLS mut^ was constructed by substituting Arg214/215 with Ala214/215 in the NLS of STAT3, and the mutant was confirmed by sequencing (GENEWIZ, Suzhou, China). siRNAs specific to STAT3 were purchased from RiboBio Co. (Guangzhou, China). Transfection of plasmids or siRNAs was performed with Lipofectamine 3000 (Life Technologies) according to the manufacturer’s instruction.

Mouse anti-VP1 (ab36367) anti-MX1 (ab95926) and anti-total STAT3 (9139T) antibodies were purchased from Abcam (Cambridge, UK). Rabbit anti-VP1 antibody (GTX132338) was purchased from GeneTex (Irvine, CA, USA). Anti-pSTAT3^705^ (sc-8059) and anti-pSTAT3^727^ (sc-136193) antibodies were purchased from Santa Cruz Biotechnology. Monoclonal antibodies for STAT3(#12640), pSTAT1^701^(#7649), and pSTAT2^690^(#4441) were obtained from Cell Signaling Technology (Beverly, MA, USA). Anti-eIF4G (07-1800) antibody was obtained from Millipore (Billerica, MA, USA). Antibodies specific for unSTAT3 (10253-2-AP), KPNA1 (18137-1-AP), GAPDH (10494-1-AP), β-actin (60008-11-Ig), ubiquitin (10201-2-AP), OAS1 (14955-1-AP), and flag (20543-1-AP) were purchased from Proteintech (Rosemont, IL, USA). IRDye 680 goat-anti-rabbit and IRDye 800 goat-anti-mouse were purchased from Li-COR (Lincoln, NE, USA). Proteasome inhibitor epoxomicin (S7038) and STAT3 inhibitors Stattic (S7024) were purchased from Selleckchem (Houston, TX, USA). The cytotoxicity of different doses was pretested, and the working concentrations were chosen to make sure the cell viability was not affected. 

### 4.4. Western Blot

Cells were lysed with RIPA lysis buffer (Santa Cruz Biotechnology) and total protein concentrations were determined by the BCA protein assay kit (Pierce, Rockford, IL, USA). The harvested protein was subjected to SDS polyacrylamide gel electrophoresis (SDS-PAGE) and transferred to polyvinylidene difluoride (PVDF) membranes (Millipore, Billerica, MA, USA). After being blocked by Odyssey Blocking buffer (Li-COR), the membranes were incubated with appropriate primary and secondary antibodies, and then visualized on a Li-COR Odyssey Infrared Imager.

### 4.5. PCR Array and qRT-PCR Analysis

Total RNAs were extracted from T98G cells at indicated time points post EV71 infection. After reverse-transcription, the cDNA was subjected to PCR Array analysis (Wcgene Biotech, Shanghai, China). The genes with fold changes are listed in the supplementary material (Table S1). Quantitative real time-PCR (qRT-PCR) was performed using the ABI SYBR Green Master Mix (Life Technologies) followed by detection on an ABI Prism 7500 Sequence Detection System. GAPDH was used for normalization of mRNA, and analysis was carried out using the 2-^ΔΔCt^ method. The sequences of the primer pairs are listed in the supplementary material (Table S2).

### 4.6. ELISA Assay

INF-α and INF-β in the cell supernatant were detected using ELISA kits following the manufacturer’s instruction (EK Bioscience, Shanghai, China). Briefly, 96-well plates were coated with 50 μL capture antibodies overnight. Plates were washed and 100 μL cleared supernatants were added; after incubation and PBST washing, diluted detection antibody was added into each well and incubated for 1 h. After washing, diluted HRP conjugate was added to each well for 30 min, followed with the substrate to develop a color reaction by incubating the plates in the dark for 30 min. The reaction was terminated by stop solution, and Optical Density was measured at 450 nm (Tecan Infinite M200Pro, Switzerland).

### 4.7. Co-Immunoprecipitation Assay

Cells were lysed with NP40 lysis buffer. Antibodies were diluted in 200 μL PBS with Tween-20 and then added to 1.5 mg Magnetic Dynabeads Protein G (Invitrogen); the mix was incubated with rotation for 15 min. Non-specific IgG was used as an isotype control. After separating the beads from the solution by a magnet, cell lysates were added to the beads and incubated with rotation. The precipitates were washed 3 times and subjected to western blot analysis.

### 4.8. Luciferase Assay

For ISRE reporter activity detection, pISRE-Luc and a transfection control pRL-TK were co-transfected with STAT3-specific siRNAs into T98G cells; 48 h post the transfection, the cells were either infected with EV71 for 24 h or treated with 20 ng/mL IFN-β for 12h, and cell lysates were prepared and subjected to dual luciferase activity analysis by the Dual Luciferase Assay System (Promega). The data were normalized relative to pRL-TK values.

### 4.9. Immunofluorescence Staining and Confocal Microscopy

Cells were fixed with 4% paraformaldehyde and permeabilized with 0.5% Triton X-100 for 15 min. After being washed with PBS and blocked with BSA, the cells were incubated with primary antibodies for 2 h followed by secondary antibodies for 50 min. The nucleus was stained with DAPI followed by thorough washing. Slides were monitored by Olympus FluoView FV10i confocal microscope (Tokyo, Japan).

### 4.10. Statistical Analysis

Statistical analysis was performed by unpaired t-test or one-way ANOVA with Tukey’s multiple comparisons using GraphPad Prism software. The data are presented as the mean ± standard deviation (S.D.). * *p* < 0.05, ** *p* < 0.01, *** *p* < 0.001. 

## Supplementary Materials

Supplementary materials can be found at https://www.mdpi.com/1422-0067/20/19/4870/s1.

## Abbreviations

EV71	Enterovirus 71
STAT	Signal transducer and activator of transcription
HFMD	Hand, foot and mouth disease
KPNA1	Karyopherin-α1
PAMPs	Pathogen-associated molecular patterns
CNS	Central nervous system
ISRE	IFN-stimulated response element
ISGs	IFN-stimulated genes
SOCS	Cytokine signaling proteins
unSTAT3	un-phosphorylated STAT3
un-phosphorylated STAT3	Epoxomicin
ME	Meningoencephalitis
FC	Febrile convulsion
JAK	Janus-Activated Kinase
CSF	Cerebrospinal fluid
TCID50	50% tissue culture infectious dose
