# Supplementary material for: STAT3 Regulates the Type I IFN-Mediated Antiviral Response by Interfering with the Nuclear Entry of STAT1
Source: Int J Mol Sci. 2019 Sep 30;20(19):4870. doi: 10.3390/ijms20194870 (PMC6801597; doi:10.3390/ijms20194870)
Supplement: Supplementary file 1 [file ijms-20-04870-s001.pdf]

STAT3 regulates type I IFN-mediated antiviral response by interfering nuclear entry of STAT1

Huanru Wang[1], Meng Yuan[1], Shuaibo Wang[4], Li Zhang[1], Rui Zhang[1], Xue Zou[1], Xiaohui Wang[5], Deyan Chen[1], Zhiwei Wu[1,2,3]*

Supporting Information list:

Fig. S1-S8

Table. S1-S2

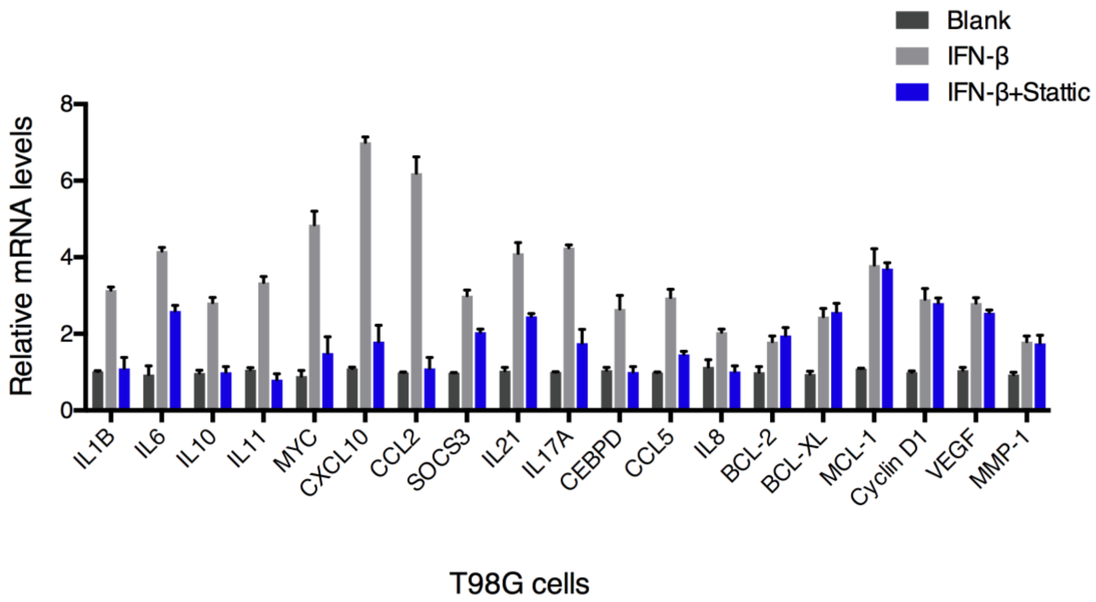

Fig S1. Verification of STAT3 target genes in T98G cells. Total RNA was extracted and reverse transcripted to cDNA. Real-time PCR was performed with specific primer sets. Genes that are upregulated by IL-6 and downregulated by Stattic were chosen as STAT3 targets for heat map analysis in Fig. 2A.

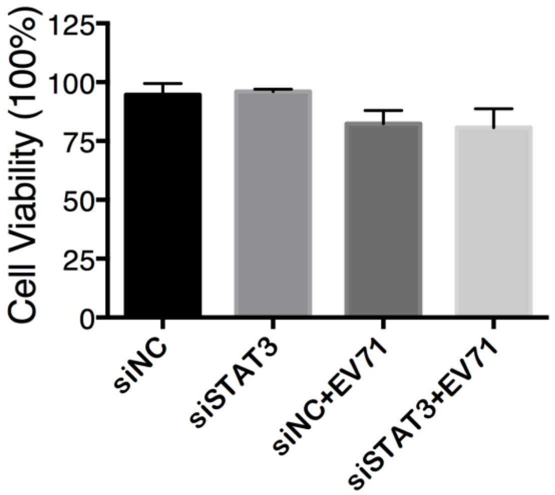

Fig S2. Cell viability was not affected by STAT3 knock down. Cell viability of T98G cells was assessed by Cell Count Kit-8. Cells were dispensed in a 96-well culture plate at 5×10^3 cells/well. Scrambled (siNC) or STAT3-specific siRNA (siSTAT3) was

transfected with Lipofectamine 3000 (Life Technologies) according to the manufacturer's instruction. 36h post transfection cells were either mock infected or infected with EV71 for 24h, 10 ul CCK-8 assay solution was added into each well, and the plate was incubated for 2 h at 37 °C. The absorbance at 450 nm was then measured, and cell viability was plotted as the percent viable cells of the siNC-transfected control cells.

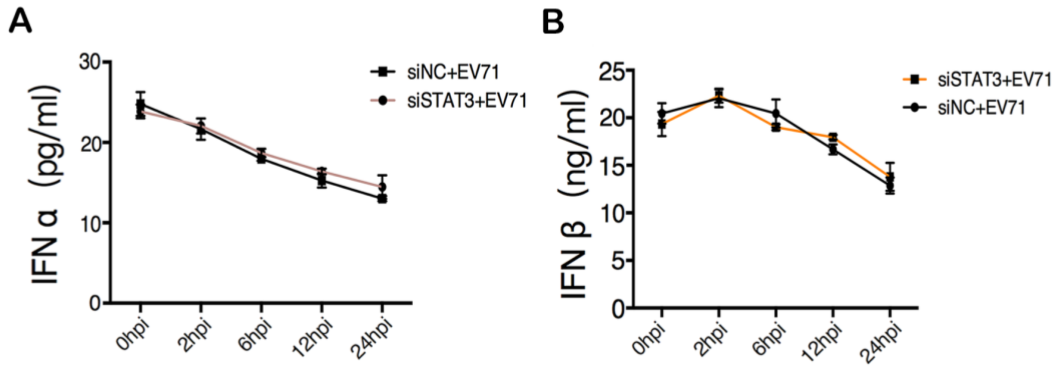

Fig S3. STAT3 knockdown did not change the expression patterns of IFN-α and IFN-β upon EV71 infection. T98G cells were transfected with STAT3 specific siRNA for 48h, cell culture supernatant was harvested at indicated time points post EV71 infection. Expression levels of IFN-α **(A)** and IFN-β **(B)** were analyzed by ELISA.

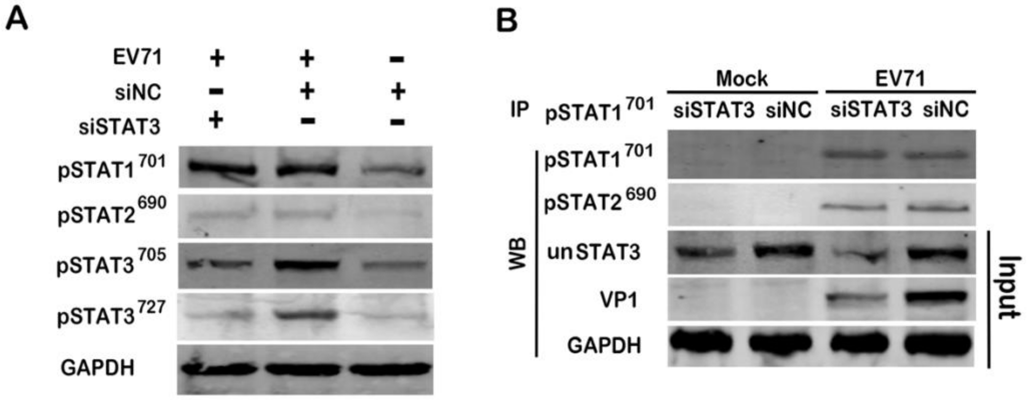

Fig S4. Phosphorylation and dimerization of STAT1 were not affected by STAT3 knockdown. (A) After STAT3 siRNA transfection and EV71 infection, T98G cells were harvested and protein levels of phosphorylated STAT1, STAT2 and STAT3 were analyzed by western blot. **(B)** The lysates of EV71 infected or mock infected STAT3 KD T98G cells were subjected to immunoprecipitation (IP) assay with antibody specific to Tyr701 phosphorylated STAT1(pSTAT1$_{701}$), and the precipitated complex was analyzed by western blot with indicated antibodies.

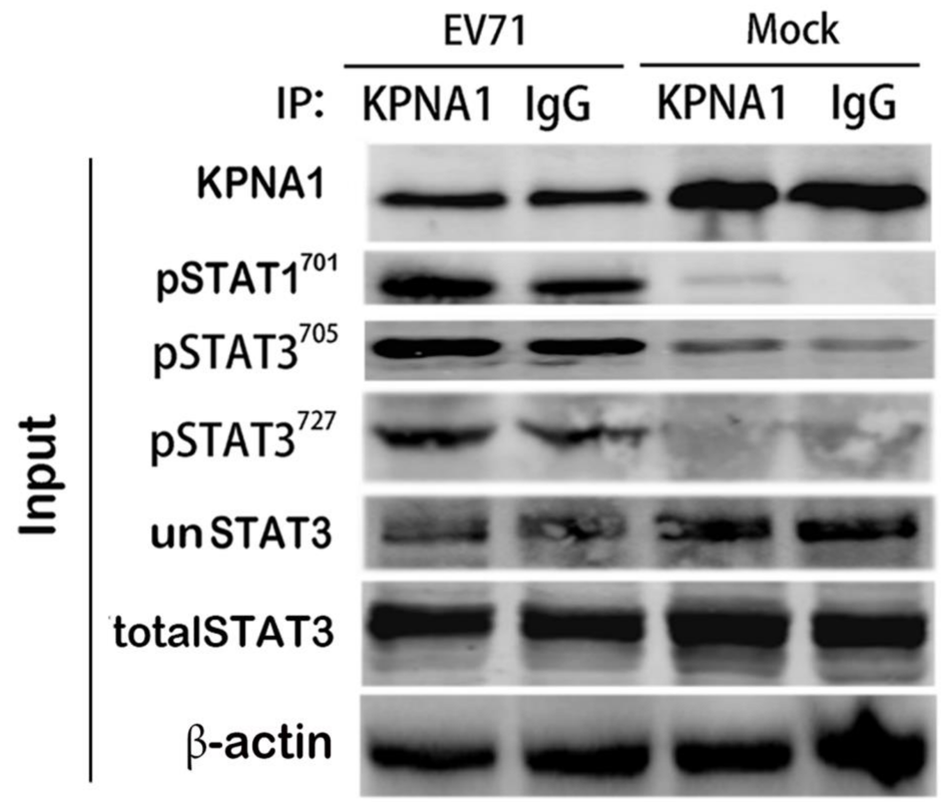

Fig S5. Expression levels of indicated proteins in input protein samples in IP assay. Total protein of T98G cells were harvested at 24h post infection. Before immunoprecipitation, 60ug total protein samples were taken from each group and western blot were performed with specific antibodies to determine expression levels of indicated proteins.

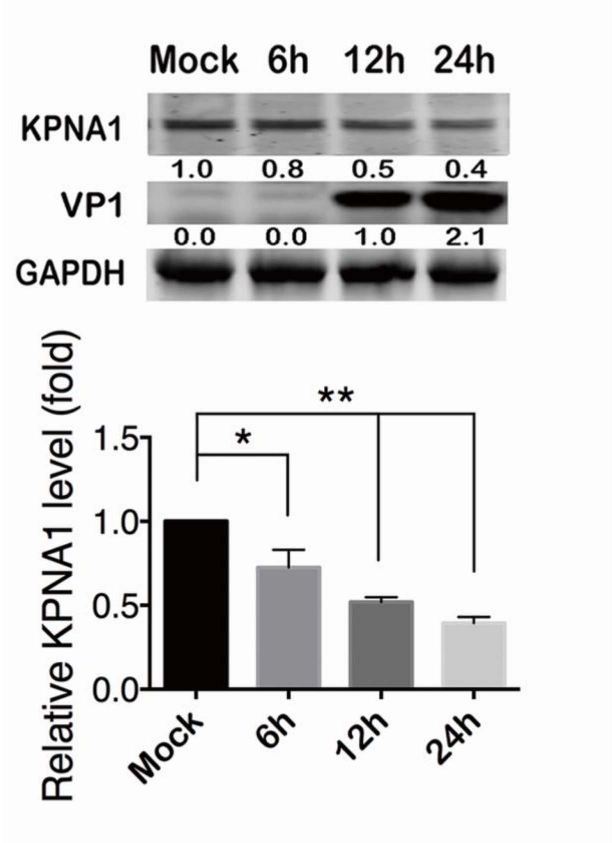

Fig S6. EV71 infection induced KPNA1 degradation in T98G cells. KPNA1 protein post EV71 infection was analyzed by western blot. Relative expression level of KPNA1 was calculated.

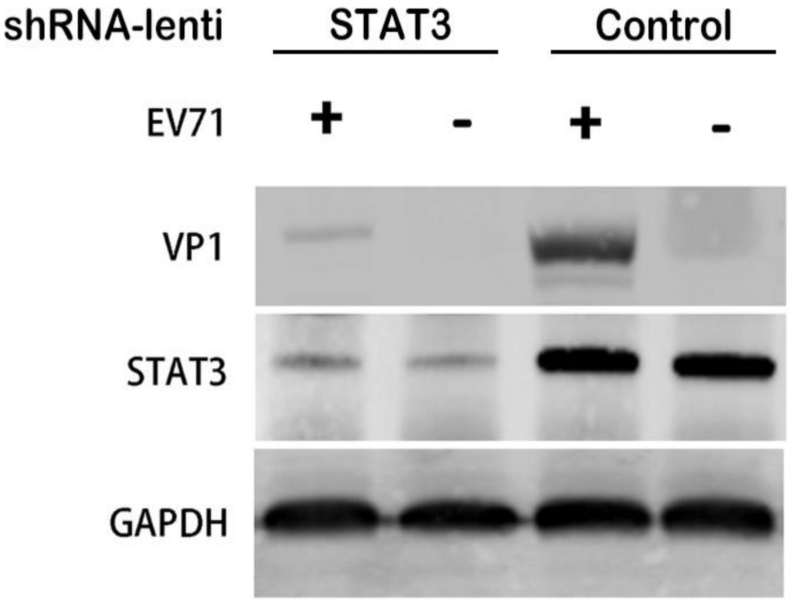

Fig S7. EV71 replication was restricted in T98G cells infected by shSTAT3-lentivirus. 1.0 × 10^7 infectious units of lentiviral particles were added in to T98G cells for 60h, then the cells were mock infected or infected with EV71 at the moi of 5, expression levels of total STAT3 and VP1 were analyzed by western blot at 36hpi.

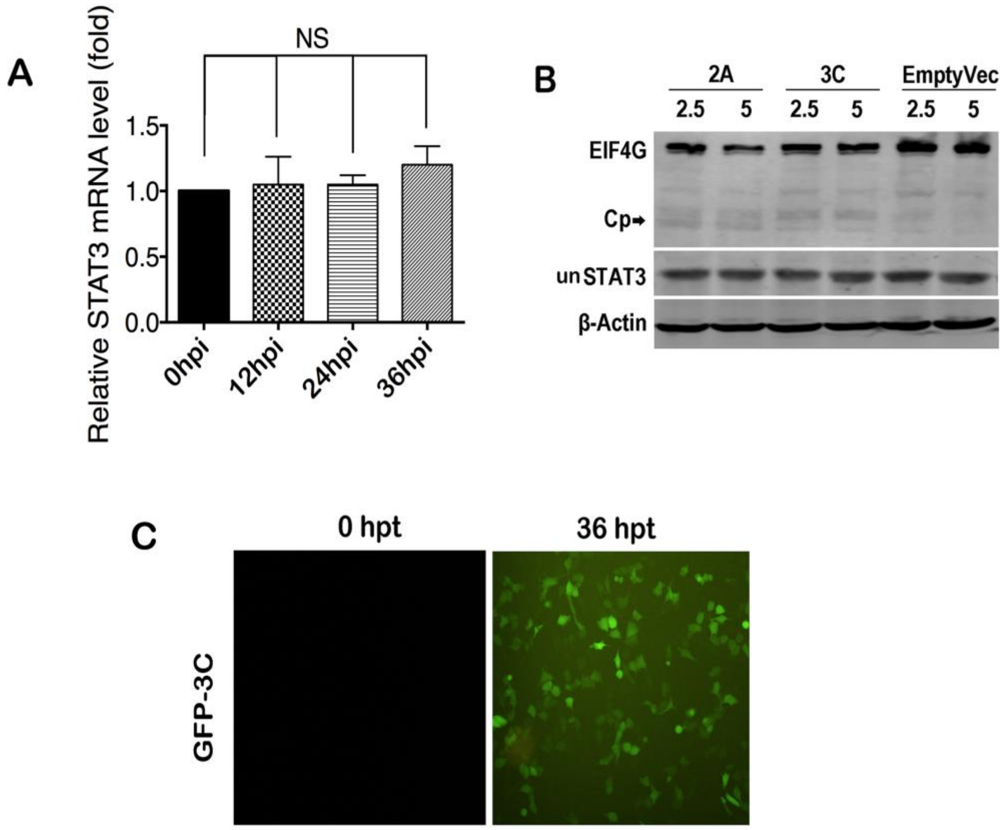

Fig S8. EV71 2A or 3C transfection did not affect unSTAT3 protein level. (A) STAT3 mRNA levels at indicated time points post infection were determined by real-time PCR. **(B)** After 2A and 3C transfection of T98G cells for 48h, unSTAT3 protein level was analyzed. Transfection efficiency and activity of 2A were indicated by EIF4G degradation. **(C)** Transfection efficiency of 3C in Fig. 7C was determined by GFP expression.

Table S1. Fold changes of the tested genes in PCR Array analysis.

Target Name	2h/mock	6h/mock	12h/mock	24h/mock
AKT1	17.89491122	20.5130254	12.64445455	13.66251007
BAX	3.760671881	6.519654221	3.829498245	3.387683023
CCL2	3.535176413	1.624029462	0.500403601	0.187342298
CCL5	0.415940653	0.820068147	0.861393254	1.869789612
CDC25A	70.59499044	78.13347635	51.97789565	147.4169294
CDKN1A	6.15120359	5.597576521	3.562108629	4.514403607
CEBPD	0.20019682	0.197779552	0.078515636	0.017573752
IL10	5.148950125	5.668672749	0.319322092	0.58241869
CXCL10	23.07277445	29.41088076	0.987204943	0.695769898
CXCL12	3.168684641	2.990481093	2.010252987	1.427658889
CXCR4	20.54186983	26.12015511	38.94836955	86.35269165
EGFR	5.257000363	5.133552081	2.839133513	6.108313814
FAS	3.571221169	3.040511119	1.404298985	0.880554786
IL11	3.119446652	2.215110041	2.157354728	35.84415184
IL12A	1.552228037	1.798471168	1.171719208	1.269755981
IL17A	0.047949663	0.072568266	0.03428241	0.790194354
IL18RA	0.03321231	0.041321241	0.031332241	0.051341251

IL1B	2.589385387	3.249896949	1.448856989	0.309677084
IL1R1	0.001909967	0.001372916	0.001307189	0.000537823
IL21	0.145701036	0.213210367	0.118835088	0.189517821
IL23A	0.371135364	0.634926186	0.347966295	0.785884165
IL2RA	0.3864598	0.295645935	0.028132981	0.2964798
IL6	4.075210429	2.339076969	1.381197359	0.390545739
IL6ST	1.422390426	0.880550087	0.39163478	0.441479907
IL8	1.340064068	0.833423313	0.978740666	0.520714607
JAK2	1.287135253	1.35393302	0.511416417	0.581675398
LIFR	0.469868501	0.262809153	0.198436419	0.1875306
MAP2K1	3.988256705	4.148735435	2.640034845	4.608342779
MAPK1	9.536413173	10.69880459	5.659863712	6.643370621
MAPK14	18.78535638	20.53459144	10.7931282	14.78511106
MAPK3	4.385924519	5.852940277	3.698253579	1.940338895
MAPK8	6.955207882	9.338276124	5.733555396	6.652070324
MET	16.22861598	12.53746672	6.25597103	9.235083603
MTOR	9.408520605	10.09938659	6.512236444	8.872219256
MYC	6.316368593	7.399235201	4.464050088	7.985426288
OSMR	2.288113799	2.555331743	1.468383014	2.710774146
PIAS3	5.169179983	6.598387181	3.608048316	6.956472765
PIM1	1.37220378	1.609064829	1.166237077	1.34146432
RAC1	2.432166538	2.550236206	1.318092657	1.139833984

SOCS1	1.922249691	3.411910089	3.237745924	2.629684273
SOCS3	0.099386305	0.28323545	0.26895954	0.304773037
SRC	6.292955478	7.994512882	4.615003755	7.167481169
TNFRSF10B	6.902929939	6.736796611	3.803790713	5.019531472
TNFRSF1A	8.42884641	9.324765894	5.581113165	6.982592627
TNFRSF1B	#DIV/0!	0.290209688	0.165503381	0.277998357

Table S2. Primer sets used in PCR array and qRT-PCR analysis.

CXCL12 (SDF1)	ATTCTCAACACTCCAAACTGTGC	ACTTTAGCTTCGGGTCAATGC
EGFR	TTGCCGCAAAGTGTGTAACG	GTCACCCCTAAATGCCACCG
IL10	GACTTTAAGGGTTACCTGGGTTG	TCACATGCGCCTTGATGTCTG
IL11	CGAGCGGACCTACTGTCCTA	GCCCAGTCAAGTGTCAGGTG
IL17A	AGATTACTACAACCGATCCACCT	GGGGACAGAGTTCATGTGGTA
IL21	TAGAGACAAACTGTGAGTGGTCA	GGGCATGTTAGTCTGTGTTTCTG
IL23A	CTCAGGGACAACAGTCAGTTC	ACAGGGCTATCAGGGAGCA
IL6	ACTCACCTCTTCAGAACGAATTG	CCATCTTTGGAAGGTTCAGGTTG
CD4	TGCCTCAGTATGCTGGCTCT	GAGACCTTTGCCTCCTTGTTC
CD40 (TNFRSF5)	TTGGGGTCAAGCAGATTGCTA	GCAGATGACACATTGGAGAAGA
CD80	AAACTCGCATCTACTGGCAAA	GGTTCTTGTACTCGGGCCATA
CSF3R (GM-CSFR)	GCGCGAGCAATAGCAACAAG	GTCACGATGATCTCATAGAGCTG
CXCR4	ACTACACCGAGGAAATGGGCT	CCCACAATGCCAGTTAAGAAGA
FAS (TNFRSF6)	AGATTGTGTGATGAAGGACATGG	TGTTGCTGGTGAGTGTGCATT
IL18R1	CCTTGACCCTTTGGGTGCTTA	CTCATGTGCAAGTGAACACGA
IL1R1	GGCTGAAAAGCATAGAGGGAAC	CTGGGCTCACAATCACAGG
IL2RA (CD25)	CGCAGAATAAAAAGCGGGTCA	ACTTGTTTCGTTGTGTTCCGA
IL6R	CCCCTCAGCAATGTTGTTTGT	CTCCGGGACTGCTAACTGG
IL6ST (GP130)	CGGACAGCTTGAACAGAATGT	ACCATCCCACTCACACCTCA
LIFR	TGGAACGACAGGGGTTCAGT	GAGTTGTGTTGTGGGTCACTAA
OSMR	AATGTCAGTGAAGGCATGAAAGG	GAAGGTTGTTTAGACCACCCC
TNFRSF10B (DR5)	GCCCCACAACAAAAGAGGTC	AGGTCATTCCAGTGAGTGCTA
TNFRSF1A	TCACCGCTTCAGAAAACCACC	GGTCCACTGTGCAAGAAGAGA
TNFRSF1B	TTCATCCACGGATATTTGCAGG	GCTGGGGTAAGTGTACTGCC
AKT1	AGCGACGTGGCTATTGTGAAG	GCCATCATTCTTGAGGAGGAAGT
JAK2	TCTGGGGAGTATGTTGCAGAA	AGACATGGTTGGGTGGATACC

JAK3	CTGCACGTAGATGGGGTGG	CACGATCAGGTTGGACTTTTCT
MAP2K1 (MEK1)	CAATGGCGGTGTGGTGTTC	GATTGCGGGTTTGATCTCCAG
MAPK1 (ERK2)	TCACACAGGGTTCCTGACAGA	ATGCAGCCTACAGACCAAATATC
MAPK14	TCAGTCCATCATTCATGCGAAA	AACGTCCAACAGACCAATCAC
MAPK3 (ERK1)	ACTCCAAAGCCCTTGACCTG	GGACTGGCCCACCTCATC
MAPK8 (JNK1)	TCTGGTATGATCCTTCTGAAGCA	TCCTCCAAGTCCATAACTTCCTT
MTOR	TCCGAGAGATGAGTCAAGAGG	CACCTTCCACTCCTATGAGGC
MYC	TCCCTCCACTCGGAAGGAC	CTGGTGCATTTTCGGTTGTTG
PIAS3	CTGGGCGAATTAAAGCACATGG	AAAGCGTCGTCGGTAAAGCTC
RAC1	TACGCCCCCTATCCTATCCG	CAATCGGCTTGTCTTTGCCC
SOCS1	TTTTCGCCCTTAGCGTGAAGA	GAGGCAGTCGAAGCTCTCG
SOCS3	GGGGAGTACCACCTGAGTCT	TGTGGTTGCTATCGTCCCAC
SRC	GACAGGCTACATCCCCAGC	CGTCTGGTGATCTTGCCAAAA
TYK2	GAACCGGCTGTGTACCGTT	ACGTCATTCACAAACTCATGCTT
BAX	CCCGAGAGGTCTTTTTCCGAG	CCAGCCCATGATGGTTCTGAT
BCL2	GGTGGGGTCATGTGTGTGG	CGGTTCAGGTACTCAGTCATCC
CDC25A	GTGAAGGCGCTATTTGGCG	TGGTTGCTCATAATCACTGCC
CDKN1A	CGATGGAACTTCGACTTTGTCA	GCACAAGGGTACAAGACAGTG
HGF	GCTATCGGGGTAAAGACCTACA	CGTAGCGTACCTCTGGATTGC
MET	AGCAATGGGGAGTGTAAAGAGG	CCCAGTCTTGTACTCAGCAAC
PIM1	GAGAAGGACCGGATTTCCGAC	CAGTCCAGGAGCCTAATGACG
STAT3	ACCAGCAGTATAGCCGCTTC	GCCACAATCCGGGCAATCT
CCL2 (MCP-1)	CAGCCAGATGCAATCAATGCC	TGGAATCCTGAACCCACTTCT
CCL5 (RANTES)	CTGCTTTGCCTACATTGCCC	TCGGGTGACAAAGACGACTG
CEBPD	GGAGAGACTCAGCAACGACC	TTGCGCTCCTATGTCCCAAG
CXCL10 (INP10)	GTGGCATTCAAGGAGTACCTC	TGATGGCCTTCGATTCTGGATT
IL12A	CCTTGCACTTCTGAAGAGATTGA	ACAGGGCCATCATAAAAGAGGT
IL1B	ATGATGGCTTATTACAGTGGCAA	GTCGGAGATTCGTAGCTGGA
IL8	ACTGAGAGTGATTGAGAGTGGAC	AACCCTCTGCACCCAGTTTTC
GAPDH	ACAACTTTGGTATCGTGGAAGG	GCCATCACGCCACAGTTTC
